# Genomic Surveillance of SARS-CoV-2: Data Analysis and Assessment of Tunisian Strategy from January 2021 to February 2022

**DOI:** 10.3390/epidemiologia5010005

**Published:** 2024-02-06

**Authors:** Arwa Neffati, Mouna Safer, Wissal Kalai, Aicha Hechaichi, Sonia Dhaouadi, Hajer Letaief, Chaima Aichouch, Leila Bouabid, Sondes Darouiche, Nawel El Mili, Henda Triki, Ilhem Boutiba, Maha Mastouri, Lamia Fki Berrajah, Nissaf Bouafif Ben Alaya

**Affiliations:** 1 National Observatory of New and Emerging Diseases, Tunis 1002, Tunisia; safermouna@gmail.com (M.S.); kalai_wissal@yahoo.com (W.K.); aicha.hechaichi@gmail.com (A.H.); sonidhaouadi88@gmail.com (S.D.); hejerletaief@gmail.com (H.L.); ch.aich1996@gmail.com (C.A.); leila.bouabid@gmail.com (L.B.); drsondesderouiche@gmail.com (S.D.); elmilinawel1994@gmail.com (N.E.M.); nissafba@yahoo.fr (N.B.B.A.); 2 Faculty of Medicine of Tunis, University of Tunis El Manar, Tunis 1006, Tunisia; henda.triki@pasteur.tn (H.T.); ilhem.boutiba@gmail.com (I.B.); 3 Pasteur Institute Tunis, Tunis 1002, Tunisia; 4 Microbiology Laboratory, Charles Nicole Hospital, Tunis 1938, Tunisia; 5 Laboratory of Microbiology, Fattouma Bourguiba Hospital, Monastir 5000, Tunisia; mastourimaha@yahoo.fr; 6 Faculty of Pharmacy of Monastir, University of Monastir, Monastir 5000, Tunisia; 7 Laboratory of Microbiology, Habib Bourguiba, Sfax 3029, Tunisia; lamiasfax2003@yahoo.fr; 8 Faculty of Medicine, University of Sfax, Sfax 3029, Tunisia

**Keywords:** SARS-CoV-2, Public Health Surveillance, sequence analysis, RNA, SARS-CoV-2 variants, internal audit, Tunisia

## Abstract

Due to the emergence of the SARS-CoV-2 B.1.1.7 (Alpha) variant in the UK in 2020 and its risk of increased transmission, the Ministry of Health in Tunisia implemented a sequencing surveillance strategy for SARS-CoV-2. The aim of this study was to analyze SARS-CoV-2 genomic surveillance data in Tunisia (January 2021–February 2022) and to assess the implementation of the sequencing strategy for SARS-CoV-2 in accordance with national recommendations and the guidance for SARS-CoV-2 genomic surveillance for public health goals. A descriptive study of all sequenced RT-PCR samples sequenced (January 2021–February2022). An internal audit was also done to assess the compliance against standards covering national recommendations and the Guidance for SARS-CoV-2 genomic surveillance for public health goals. A total of 12 simple or composite requirements related to the following areas were included in the audit standards: sampling (one requirements); data collection/analysis (six requirements); partnership (one requirement); and ethical considerations (one requirement). A total of 4819 samples were sent to laboratories and 4278 samples were sequenced. A total of 3648 samples were classified. Positive variants of concern (VOC) samples were 80.92%, differentiated as follows: Alpha, 40.24%; Beta, 0.24%; Gamma, 0.03%; Delta, 45.26%; and Omicron, 14.19%. Three principal phases of VOCs per ISO-week were shown: Alpha 3/2021–25/2021; Delta 26/2021–2/2022; and Omicron 3/2022–6/2022. Levels of compliance were identified; from a total of 12 requirements, 7 were considered as “not met”, 4 as “partially met”, and 1 as “fully met” but including not totally achieved objectives. In conclusion, the internal audit of the national SARS-CoV-2 sequencing strategy revealed an overall “not met” level of compliance. The results offered a trigger to collaborate with all stakeholders to develop a surveillance strategy for early detection and response to outbreaks caused by VOCs.

## 1. Introduction

The end of 2020 was marked by the emergence of SARS-CoV-2 variants that presented increased public health risks on a global scale. As of November 2020, nearly 46 million cases and 1.2 million deaths have been reported globally [1,2]. Based on the potential impact of these variants on transmissibility, severity, clinical presentation, and the effectiveness of control and prevention measures (diagnostic tools, vaccination, therapeutic molecules), they have been classified by the World Health Organization (WHO) as variant of concern (VOC), variant of interest (VOI), or variant under monitoring (VUM) [1]. 

Genomic sequencing activity directed at pathogen surveillance has shown, over the last decade, its contribution to the detection and control of infectious disease outbreaks, facilitating the production of diagnostics, drugs, and vaccines, and guiding response activities [3]. The emergence of SARS-CoV-2 has further increased the importance of genomic surveillance data, with 4.8 million genomes deposited in the Global Initiative on Sharing All Influenza Data (GISAID) through 31 October 2021. Genomic surveillance has been very important to the early detect the mutations, to monitor the virus evolution, and to evaluate the degree of similarities between the different variants with vaccine strains [4].

This SARS-CoV-2 genomic surveillance activity is a critical public health function. It facilitated research activities prioritization and guided informed response decisions which reflected the efficiency of investing in building capacities in this area [5].

Implementation of genomic surveillance is cheaper and easier than before, but its large-scale implementation is still challenging, especially in low-income countries [6]. Genomic surveillance is generally based on two complementary approaches: the first is to collect a representative sample of the confirmed COVID-19 cases; and the second is to apply a systematic identification of any phenotypic change in the virus genomic sequence or importation of a VOC from other country. Data from both approaches will be useful to generate indicators to better understand the evolution of the virus and its potential impact and to guide public health actions [7].

The Alpha variant emerged in the UK in December 2020 [8]. Alpha variant has a higher infectivity rate, so the risk of virus importation was higher. As a response to its emergence, the Ministry of health of Tunisia implemented a SARS-CoV-2 sequencing strategy. A committee was established of all stakeholders including the Ministry of Health, the National Observatory of New and Emerging Diseases, the virology laboratories involved in sequencing activities, and the Medical Biology Laboratory Unit. The recommendations and guidelines for sequencing SARS-CoV-2 for the purpose of Public Health Surveillance, which were prepared by the WHO, United States Centers for Disease Control and Prevention (CDC), African CDC, European CDC, and expert advisory groups (e.g., WHO Technical Advisory Group on Epidemiology), were summarized and adapted to national sequencing capacities by the “SARS-CoV-2 Sequencing Expert Group”. Accordingly, a national strategy for surveillance of SARS-CoV-2 variants of concern was developed and shared among stakeholders at central and regional levels. Updates of this strategy according to the evolution of the different phases of the epidemic, the emergence of new variants, and national capacities, as well as the possibilities of reinforcement by accessing international sequencing partnerships, were subsequently carried out.

The ongoing global circulation of SARS-CoV-2 and repeated emergence of new variants indicate the need for robust genomic surveillance to inform public health responses. Sampling bias or systematic differences in sample characteristics between COVID-19 cases with sequenced specimens and total COVID-19 cases is a concern and might produce inaccurate representations of variant distribution within the population.

To ensure generalizability and equity when using genomic and epidemiologic data for public health purposes, the methods for genomic surveillance must ensure a representative sample from the population of interest. In Tunisia, the implementation and application of this genomic surveillance in accordance with the national recommendations and the requirements of SARS-CoV-2 genomic surveillance for public health goals has encountered many difficulties. In fact, there were difficulties in managing missing data, making it impossible to characterize phenotypically the different variants. Moreover, we can mention the non-application of targeted and random sampling procedures causing bias in monitoring the proportional circulation of the different variants.

Thus, this study aimed to analyze the SARS-CoV-2 genomic surveillance data in Tunisia from January 2021 to February 2022 and to assess the implementation of the SARS-CoV-2 sequencing strategy in accordance with national recommendations and the guidance for SARS-CoV-2 genomic surveillance for public health goals. 

## 2. Materials and Methods

### 2.1. Study Design

To meet the two objectives of this work, a descriptive analysis was conducted on all RT-PCR sequenced SARS-CoV-2 genomic surveillance data from January 2021 to February 2022. Additionally, to evaluate the sequencing strategy, an internal audit assessing compliance with the implementation of the strategy was carried out compared to a reference framework which included the procedures of the national strategy and the requirements of SARS-CoV-2 genomic surveillance for public health purposes.

### 2.2. Study Population

We included all confirmed COVID-19 cases (SARS-CoV-2 RNA detected by molecular amplification) sequenced and reported to the National Observatory of New and Emerging Diseases from January 2021 through February 2022.

### 2.3. Genomic Surveillance System Design

In January 2021, the National Observatory of New and Emerging Diseases (with five reference SARS-CoV-2 laboratories) established a surveillance strategy to monitor the genomic epidemiology of SARS-CoV-2 in Tunisia. Partner laboratories were selected to maximize geographic coverage and specimen numbers. The initial proportion of randomly selected positive specimens submitted for sequencing was designed to balance geographic coverage regionally and match available sequencing capacity.

Specimens specifically selected for targeted sequencing as part of outbreak investigations because of travel history, known vaccine breakthrough status, or spike gene target failures were also selected outside the random selection process.

### 2.4. Audit Standards of the Tunisian Sequencing Strategy for SARS-CoV-2

The “audit standards” were designed by compiling the guidance for SARS-CoV-2 genomic surveillance for public health goals. A total of 12 requirements, distributed over five areas, were included in the audit standards, as shown in Table 1.

### 2.5. Data Collection

The main sources of data were the national SARS-CoV-2 databases, including “genomic surveillance” and “Confirmed cases”. The variables extracted from the databases included demographic data (date of birth, sex, origin), the date of confirmation of COVID-19 infection, sequencing method(s), and the SARS-CoV-2 variant. The variables of interest in the different databases were cross-referenced and checked against the sequenced patient data.

### 2.6. Representativeness and Timeliness Evaluation

We assessed representativeness of genomic surveillance data by comparing COVID-19 cases with sequenced specimens to all COVID-19 cases during the same period according to sex and age; we represented the percentage of cases with sequenced specimens by governorate and month to visualize spatiotemporal sampling. We evaluated areas with high sequencing coverage to determine geographic representativeness.

We assessed timeliness of data by comparing the interval between initial specimen collection and genomic data transmission to the national observatory of New and Emerging Diseases. We assessed median timeliness by month and compared data transmitted within <14 days, 14–27 days, and >28 days after specimen collection.

### 2.7. Ethical Approval

This study included surveillance activity and was exempt from review and informed consent. Confidentiality of individuals was respected during data analysis and reporting. 

### 2.8. Analysis 

#### 2.8.1. Analysis of SARS-CoV-2 Genomic Surveillance Data

Data were entered into MS Excel and analyzed using R Software. The following indicators were calculated: number of the total sample to be sequenced, number and proportion of the sequenced sample, number and proportion of classified variants, and proportional distribution of classified variants. Data were described as frequencies and percentages (N, %), and the evolution of the sequencing activity per ISO week and the evolution of the proportional distribution of VOCs per ISO week were presented graphically. 

#### 2.8.2. Evaluation of the National Sequencing Strategy

A qualitative assessment for compliance to different standards was done and the level of compliance was identified to be one of the following:

- If there is total lack of required information or activities, the level of compliance is considered as “not met”;
- If required information or activities are partially respected, the level of compliance is considered as “partially met”;
- If we disposed of all the information needed and the activity was well realized, the level of compliance is considered as “fully met”.

## 3. Results

### 3.1. Surveillance Sequencing Data of SARS-CoV-2

**Figure 1:**
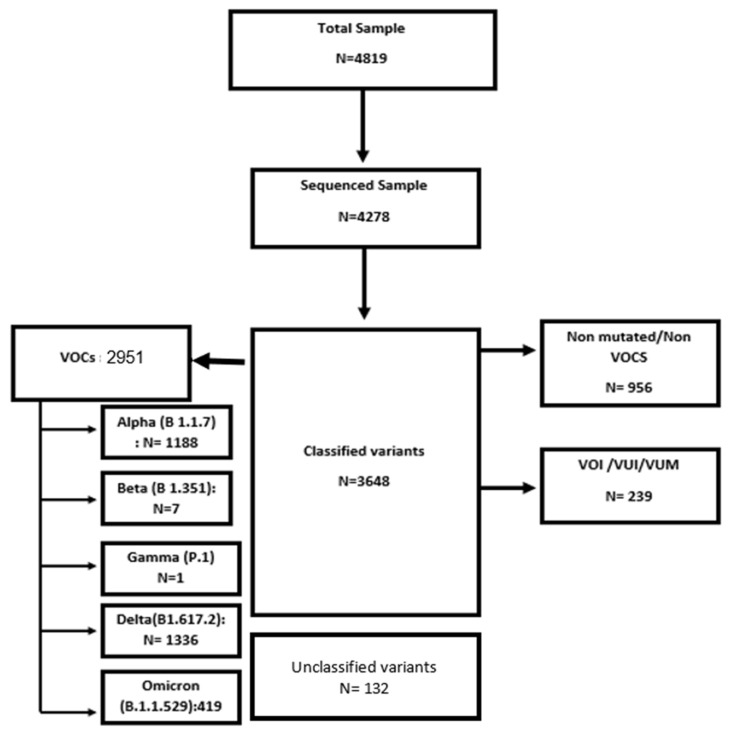
Sequenced and classified sample of RT-PCR: surveillance variants of concern of SARS-CoV-2, Tunisia, from January 2021 to February 2022.

A total of 4819 samples were sent to sequencing laboratories during the study period and 4278 (75.7%) samples were sequenced. Of those, 85.2% of samples were classified. Positive VOC samples came to 80.9%, differentiated as Alpha (1187.88, 40.24%); Beta (7.085, 0.24%); Gamma (0.886, 0.03%); Delta (1336.07, 45.26%); and Omicron (419, 14.19%) (Figure 1).

The sequencing methods used are reported in Table 2. Screening was the method used for the majority, followed by partial genomic sequencing.

**Figure 2:**
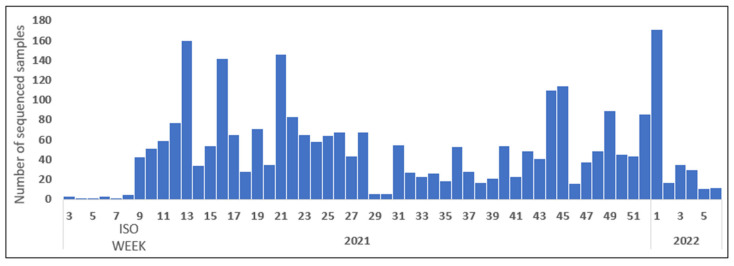
Evolution of SARS-CoV-2 sequencing activity in Tunisia per ISO week from January 2021 to February 2022.

**Figure 3:**
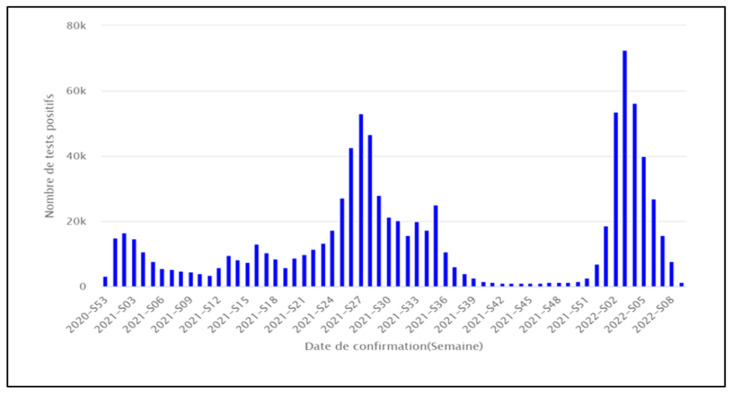
Evolution of SARS-CoV-2 confirmed cases in Tunisia per ISO week from January 2021 to February 2022. Source: National Observatory of New and Emerging Diseases.

The Sequencing activity varied throughout the period of the epidemic’s evolution, as shown in Figure 2, compared to the confirmed cases per ISO week, as shown in Figure 3.

**Figure 4:**
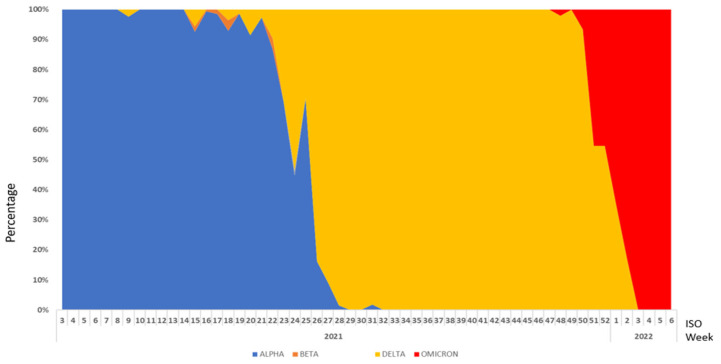
Circulation trends of VOCs per ISO week in Tunisia; from January 2021 to February 2022.

The study of the evolution of VOC’s predominance showed three phases: (1) Alpha variant predominance from ISO week 3/2021 to ISO week 25/2021; (2) Delta variant predominance from ISO week 26/2021 to ISO week 2/2022; and (3) Omicron variant predominance from ISO week 3/2022 to ISO week 6/2022 (Figure 4).

### 3.2. Results of the Internal Audit of the Tunisian Strategy of SARS-CoV-2 Genomic Surveillance

The conformity of the implementation of the national SARS-CoV-2 sequencing strategy with the requirements of the SARS-CoV-2 genomic surveillance for public health purposes is detailed in the Table 2. Of the 12 requirements; 7 were “not met”, 4 were “partially met”, and 1 was “fully met” but including not totally achieved objectives.

## 4. Discussion

This study is the first exhaustive national analysis of SARS-CoV-2 genomic sequencing surveillance data in Tunisia, with an assessment of implementation and compliance with SARS-CoV-2 genomic surveillance sequencing strategy requirements for public health purposes. By performing this evaluation, we provide information regarding the populations of sampled cases and limitations on inference affecting genomic data use. More broadly, we raise awareness regarding sampling bias in convenience-based genomic surveillance systems and support the development of robust genomic surveillance systems.

The results of the analysis of the surveillance data showed that the study of the proportional distribution of the VOCs made it possible to identify the phases of predominance and inversion of the circulation of the different VOCs. This evolution should be interpreted with caution, considering the non-representativeness of the sequenced samples throughout the evolution of the epidemic in Tunisia. A similar study has been conducted to define the relative abundance of SARS-CoV-2 lineages and to identify novel SARS-CoV-2 variants in wastewater samples from October 2020 to March 2021 in Nice. It identified a switch that occurred between January and February 2021, characterized by a rapid onset of the Alpha variant representing more than 80% of all sequences and the subsequent appearance of the delta variant.

The qualitative assessment for compliance with different standards showed that only one requirement was “fully met”, even though it included not totally achieved objectives. A study of 118 surveillance strategies of different countries based on predefined criteria showed that 45 countries (38.1% of the countries studied) had a high level of routine genomic surveillance, 17 (14.4%) had a moderate level of routine genomic surveillance, 25 (21.2%) implemented a low level of routine genomic surveillance, and 31 (26.3%) had limited genomic surveillance, such as the Eastern Mediterranean Region, followed by the African Region, the Americas, South-East Asia, and the Western Pacific Regions [9].

The non-compliant requirements identified in the evaluation of SARS-CoV-2 genomic surveillance sequencing for public health purposes are mainly related to procedure and application of random and targeted sampling; missing metadata; non-availability of match vaccine data; investigation of first cases of variants of concern; notification of variant surveillance data; rapid sharing of information; and ethical considerations.

The SARS-CoV-2 sequencing data surveillance study based on consolidated data from many sources had several struggles: difficulties in managing missing data, particularly for death and hospitalization outcomes, making it impossible to characterize the different variants phenotypically in terms of severity; moreover, the sequenced samples are not representative because of their non-compliance with sampling requirements and non-application of targeted and random sampling procedures. In fact, the bias in monitoring the proportional circulation of the different variants accounts for the impossibility pf early detection of the introduction of new variants.

The implementation of the national sequencing strategy encountered several difficulties; in particular, weekly randomly selected samples that were not sent to the laboratories in time for sequencing as, according to a study of the turnaround time (TAT, defined as the time in days between sample collection and genome submission) of SARS-CoV-2 genome sequencing across 19 geographic regions, an average of 48 days after sampling was the time required to deposit virus sequences in public databases [9].

Other epidemiologic information was of interest in assessing representativeness, including travel history, reinfection, and vaccine status. However, data for those variables were incomplete, limiting their usefulness. The full potential of genomic epidemiologic surveillance for improving public health requires pairing epidemiologic, vaccine, and clinical metadata with genomic data.

## 5. Conclusions

The sequencing of SARS-CoV-2 has made it possible to detect the introduction of variants of concern and to describe the circulation of the different viral strains throughout the evolution of the epidemic. The implementation of a sequencing strategy in line with the requirements of genomic surveillance of SARS-CoV-2 for public health purposes has encountered several problems for timely decision making. The results offer an opportunity to convene all involved stakeholders and discuss the development of an epidemiogenomic surveillance strategy for early detection and guidance as to the response measures for outbreaks caused by highly mutagenic pathogens.

## Figures and Tables

**Table 1:** The 12 requirements of the internal audit of the Tunisian SARS-CoV-2 genomic surveillance strategy, February 2022.

Areas of Requirements	Number of Requirements	Requirements
Sampling	3	1-Random samples, representative of the geographic and demographic distribution of SARS-CoV-2 infections;
		2-Targeted sampling, focusing on particular subsets of cases associated with public health risks;
		3-Outbreaks, alerts, or other unusual events.
Data collection/analysis/sharing and decision making	6	1-All reported sequences should be accompanied by metadata;
		2-Rapid sharing of genomic sequence information in public databases;
		3-Comprehensive epidemiological and clinical data can be linked to sequence information;
		4-Matching vaccine data to variant types will help determine the ability of a variant to evade the immune system response;
		5-Epidemiological studies of variants should focus on settings where the potential for generalization is high and the results are likely to be broadly relevant;
		6-The main objectives of an investigation of the first cases and their close contacts are to obtain a description or estimate of such things as the clinical picture of SARS-CoV-2 infection, and the associated disease course; the secondary infection rate (SIR) and secondary clinical attack rate of SARS-CoV-2 infection in close contacts; the serial interval of SARS-CoV-2 infection; the proportion of symptomatic cases among COVID-19 cases; and the identification of possible transmission routes.
Reporting of surveillance data on SARS-CoV-2 variants	1	Rapid sharing of information on variants is an essential element in understanding and eradicating SARS-CoV-2 globally.
Partnership	1	Countries with limited sequencing capacity are strongly encouraged to facilitate access to regional and international sequencing partnerships or to increase their capacity through existing sequencing systems or laboratory networks.
Ethical considérations	1	All ethical considerations related to genomic sequencing, sequence sharing, and related metadata will be discussed by the different stakeholders, a dossier will be prepared and submitted to an ethics committee.

**Table 2:** Conformity of the implementation of the national sequencing strategy in Tunisia with the requirements of SARS-CoV-2 genomic surveillance.

Areas of Requirements	Requirements	Level of Compliance	Problem
Sampling	1-Random samples, representative of the geographic and demographic distribution of SARS-CoV-2 infections;	Not met	Random sample was selected every week and problems in sample conservation (bank of samples) or in sample transport were detected
	2-Targeted sampling, focusing on particular subsets of cases associated with public health risks;	Partially met	Applied but in a partial way
	3-Outbreaks, alerts, or other unusual events.	Partially met	Not systematically applied
Data collection/ Analysis/ sharing and decision making	1-All reported footage should be accompanied by metadata;	Not met	Lack of information about patients personal data
	2-Rapid sharing of genomic sequence information in public databases;	Partially met	Not systematically applied
	3-Comprehensive epidemiological and clinical data can be linked to sequence information;	Partially met	Patient’s Information was not complete
	4-Matching vaccine data to variant types will help determine the ability of a variant to evade the immune system response;	Not met	We did not have any vaccination data base or information.
	5-Epidemiological studies of variants should focus on settings where the potential for generalization is high, and the results are likely to be broadly relevant;	Not met	Problem in data disponibility to conduct studies
	6-The main objectives of an investigation of the first cases and their close contacts are to obtain a description or estimate of such things as the clinical picture of SARS-CoV-2 infection, and the associated disease course; the secondary infection rate (SIR) and secondary clinical attack rate of SARS-CoV-2 infection in close contacts; the serial interval of SARS-CoV-2 infection; the proportion of symptomatic cases among COVID-19 cases; and the identification of possible transmission routes.	Not met	Lack of information
Reporting of surveillance data on SARS-CoV-2 variants	1. Rapid sharing of information on variants is an essential element in understanding and eradicating SARS-CoV-2 globally.	Not met	Lack of sharing and publications
Partnership	1. Countries with limited sequencing capacity are strongly encouraged to facilitate access to regional and international sequencing partnerships or to increase their capacity through existing sequencing systems or laboratory networks.	Fully met	There were partners, but partnership projects faced many difficulties
Ethical considerations	1. All ethical considerations related to genomic sequencing, sequence sharing, and related metadata will be discussed by the different stakeholders, a folder will be prepared and submitted to an ethics committee.	Not met	Not applied

## Data Availability

The National Observatory of New and Emerging Diseases (Tunisia), as well as the corresponding author, have full access to all data. This data is not publicly accessible but are available from the corresponding author upon reasonable request.
